# The Effects of Sunflower and Maize Crop Residue Extracts as a New Ingredient on the Quality Properties of Pork Liver Pâtés

**DOI:** 10.3390/foods13050788

**Published:** 2024-03-03

**Authors:** Milica Glišić, Marija Bošković Cabrol, Nikola Čobanović, Marija Starčević, Stevan Samardžić, Ivona Veličković, Zoran Maksimović

**Affiliations:** 1 Faculty of Veterinary Medicine, Department of Food Hygiene and Technology, University of Belgrade, 11000 Belgrade, Serbia; marija.boskoviccabrol@unipd.it (M.B.C.); cobanovic.nikola@vet.bg.ac.rs (N.Č.); 2 Department of Agronomy, Food, Natural Resources, Animal and Environment (DAFNAE), University of Padova, 35020 Padova, Italy; 3 Serbian Armed Forces, 11000 Belgrade, Serbia; marijadok@gmail.com; 4 Faculty of Pharmacy, Department of Pharmacognosy, University of Belgrade, 11000 Belgrade, Serbia; stevan.samardzic@pharmacy.bg.ac.rs (S.S.); zmaksim1@pharmacy.bg.ac.rs (Z.M.); 5 Faculty of Biology, Institute of Botany and Botanical Garden “Jevremovac”, University of Belgrade, 11000 Belgrade, Serbia; ivona@bio.bg.ac.rs

**Keywords:** agricultural waste, sunflower and maize stalks, antioxidant activity, meat emulsions, lipid oxidation, microbial quality, sensory properties

## Abstract

The present study aimed to evaluate the antioxidant capacity of ethanolic extracts from post-harvest sunflower and maize stalk residues, and their impact on the chemical composition, physicochemical parameters, lipid oxidative stability, microbiological properties, and sensory characteristics of pork liver pâtés over a 90-day storage period. Four formulations were prepared: a control group (CON), a batch with butylated hydroxytoluene as a synthetic antioxidant (BHT), 1% ethanolic extract from sunflower residues (SSRE), and 1% ethanolic extract from maize residues (MSRE). The MSRE had a higher total phenol content and showed better antioxidant activity relative to the SSRE (*p* < 0.01). The addition of SSRE decreased the lightness and increased the redness in the pork liver pâtés, with these pâtés showing the highest total color difference compared to the control (*p* < 0.01). The crop extracts increased the n-6 and total PUFA contents in pâtés and improved the PUFA/SFA ratio (*p* < 0.01). Formulations containing crop residue extracts showed higher TBARs and POV values than the control and BHT group (*p* < 0.01), indicating a pro-oxidant effect and accelerated lipid oxidation in pâtés during storage. As far as microbiological quality, the presence of crop residue extracts decreased the total viable count, lactic acid bacteria, and psychotropic aerobic bacteria (*p* < 0.01). The incorporation of crop extracts in the pork pâtés impaired their sensory quality, particularly color, odor, aroma, and flavor, and decreased their overall acceptability. These results indicated that, while the crop residue extracts were not as effective as synthetic antioxidants in preserving the lipid stability of pâtés, they demonstrated potential for enhancing the microbial quality of this type of meat product.

## 1. Introduction

Liver pâtés are traditional cooked meat products mainly manufactured from pork and veal liver, pork backfat, and other characteristic ingredients, depending on consumer habits (nuts, fruits, spices, and herbs, etc.) [1,2,3]. This type of meat product is highly appreciated by consumers all over the world due to its nutrient content, added-value ingredients, and good sensory attributes [2,4,5,6]. Liver pâtés are an important source of high-quality proteins, vitamins (B1, B12, and folic acid), and heme iron, which makes this type of meat products even more attractive, especially for children and women, who may be more susceptible to iron deficiency anemia [3,7].

Despite the presence of nitrite, sodium ascorbate, and some ingredients with proven antioxidant activity in liver pâtés, lipid oxidation during refrigerated storage has been reported as the main cause of deterioration in product quality and the development of unpleasant aromas [1,6]. This lipid oxidation limits the quality and acceptability of liver pâtés as a consequence of the degradation of polyunsaturated fatty acids and heme pigments, followed by the release of iron and alterations in texture, flavor, color, and nutritional value [4,5,8]. The high oxidative instability of liver pâtés can be ascribed to their high amount of fat (around 35%) and non-heme iron (approximately 30 mg/g of product), with the latter being considered as the major pro-oxidant compound in meat products [6]. Additionally, the manufacturing process of liver pâtés involves the chopping, mixing, and cooking of meat, fat, and liver, which increases oxidative instability and favors reactions between free fatty acids and atmospheric oxygen in the presence of catalysts such as heat and metalloproteins [3,6], where a high temperature during the heat treatment of liver pâtés reduces their activation energy for the development of oxidative reactions and leads to the decomposition of previously formed hydroperoxides [6]. Liver pâté matrix has a low level of natural endogenous antioxidants (mainly tocopherol), justifying the wide use of exogenous antioxidants, such as sodium nitrite and/or synthetic exogenous antioxidants (butylated hydroxyanisole (BHA), butylated hydroxytoluene (BHT), and propyl, octyl, and dodecyl gallates) [3,5,6,8]. However, the use of synthetic preservatives in meat products has been associated with health risks related to the carcinogenic potential of residual nitrite, N-nitrosamines, and some lipid oxidation products, and the possible hazardous effects of synthetic antioxidants on consumer health [3,5,9]. In this regard, the meat industry has been making efforts not only to directly reduce the content of potentially hazardous preservatives, but also to look for alternatives to substitute synthetic additives with food ingredients derived from natural sources that could be used as safe antimicrobials and antioxidants [3,9].

In the last decade, several studies have examined the antioxidant properties of different plant extracts (containing mainly polyphenolic compounds) as natural additives in various meat products, including grape and tea extracts in meat patties and cooked products [9], palm and persimmon by-products, and sage and rosemary essential oils in liver pâtés [3,5,6,8]. It has been demonstrated that the utilization of natural antioxidants could successfully substitute synthetic ones in meat products, whereby some of the examined extracts have approximately seven times higher antioxidant potential than BHT and BHA [5,6]. Nevertheless, natural antioxidants are usually more expensive than their synthetic counterparts and may negatively affect the color and/or flavor of the meat product [9].

A considerable amount of various plant materials generated annually, including waste from different agro-industrial sectors, such as seeds, peels, husks, leaves, and stalks, etc., or poorly harvested and discarded residues on the land, have recently gained increased attention as abundantly available and cost-effective renewable raw materials for the production of value-added compounds [10]. In this context, the European Union generates an average annual agricultural biomass production of 956 megatons of dry matter, with 442 megatons accounting for residue production; cereals contribute 74.3% and oil-bearing crops contribute 16.5% to the total agricultural waste [11]. The majority of these residues originate from maize, an economically significant crop that stands out as one of the world’s foremost staple cereals, with an annual production exceeding 1.2 billion metric tons. Simultaneously, sunflower ranks among the largest oilseed crops, surpassing 55 million metric tons per annum [12].

By-products from the agrifood industry are regarded as a good source of natural antioxidants, characterized by a substantial presence of polyphenols, which: (i) improve the technological, nutritional, and microbiological properties of meat products; (ii) provide physiological benefits for human health as they have antiviral, anti-inflammatory, anti-cancer, anti-tumor, and hepatoprotective properties; and (iii) valorize the by-product in question, which entails reductions in waste and environmental pollution, attaining the lower price of antioxidants and, consequently, increasing their profitability [10,13,14,15,16]. To the best of the authors’ knowledge, the potential of sunflower and maize agro-industrial wastes as natural sources of functional antioxidants for incorporation into the model meat-based matrix has not yet been evaluated. Considering all mentioned above, the present study aimed to determine the effects of the addition of extracts from sunflower and maize crop residue on the chemical composition, physicochemical properties, oxidative stability, microbiological status, and sensory acceptability of refrigerated stored pork liver pâtés, and to compare the observed effects with those of synthetic antioxidant (BHT) and control (without the addition of synthetic phenolic antioxidants and plant extracts) pork liver pâtés.

## 2. Materials and Methods

### 2.1. Materials and Chemicals

The sunflower and maize stalk residues were collected in October 2021 from the territory of the Autonomous Province of Vojvodina (Serbia). Butylated hydroxytoluene (≥99%, FCC, FG) was purchased from Sigma-Aldrich (Steinheim, Germany). Methanol (HPLC), ethanol (HPLC), sodium carbonate anhydrous (pro analysis), and potassium acetate anhydrous (pro analysis) were acquired from VWR Chemicals (Lutterworth, Leicestershire, UK). Glacial acetic acid (pro analysis) was purchased from Zorka Pharma (Šabac, Serbia), and sodium acetate trihydrate (pro analysis) from Centrohem (Stara Pazova, Serbia). Quercetin hydrate (HPLC) and 2,2′-azino-bis(3-ethylbenzothiazoline)-6-sulphonic acid (ABTS) (HPLC) were obtained from TCI Europe N.V. (Zwijndrecht, Belgium). 2,2-diphenyl-1-picrylhydrazyl (DPPH, HPLC), Folin-Ciocalteu reagent (pro analysis), galic acid (HPLC), iron(III) choride hexahydrate (pro analysis), iron(III) chloride heptahydrate (pro analysis), and TBA reagent (pro analysis) were acquired from Merck (Darmstadt, Germany). Potassium peroxydisulphate (pro analysis) and acetonitrile (HPLC-MS) were purchased from (ThermoFisher Scientific, Branchburg, NJ, USA). Aluminium nitrate nonahydrate (pro analysis) and 2,4,6-tris(2-piridyl)-s-triazine (TPTZ) (HPLC) were obtained from Fluka Chemie AG (Buchs, Switzerland), while formic acid (LC-MS) was acquired from Honeywell, Fluka (Seelze, Germany). For the standard solutions’ preparation, tricin (PhytoLab GmbH & Co. KG, Vestenbergsgreuth, Germany), ferulic acid (Extrasynthese, Genay Cedex, France), chlorogenic acid (Carl Roth GmbH & Co. KG, Karlsruhe, Germany), p-coumaric acid, and 3,5-di-caffeoylquinic acid (Sigma-Aldrich, St. Louis, MO, USA) were used. For fatty acid analyses, a hexane/isopropanol mixture (ASE 200, Dionex, Potsdam, Germany) and standard FAME mixture (Supelco 37 Component FAME Mix, Supelco, Bellefonte, PA, USA) were used. The media used for the cultivation of microorganisms: peptone water, plate count agar—PCA, Violet Red Bile Glucose agar—VRGB, and De Man, Rogosa, Sharp Agar-MRS were purchased from Merck (KGaA, Darmstadt, Germany).

### 2.2. Preparation of Crop Residue Extracts

The sunflower and maize stalk residue extracts were obtained by two-step extraction using hexane and 96% ethanol, as previously described by Glišić et al. [17]. Briefly, a stainless-steel extractor was loaded with 3 kg of ground plant material and extracted with a six-fold weight of hexane for 1 h at 40 °C, followed by filtration and concentration using a DLAB RE 200 Pro industrial rotary evaporator (60 °C, 60 rpm, 216–200 mbar, 150 min). The residues were then extracted with six-fold weight of ethanol/H_2_O (96:4, *v*/*v*) at 45 °C. The ethanolic extracts were further filtrated and concentrated using an evaporator under the same working conditions.

### 2.3. Evaluation of Total Phenol Content

The total phenol content in the extracts was evaluated according to Ahmad et al. [18]. Aliquots of 20 µL of the extract solution were mixed with 100 µL of Folin–Ciocalteu reagent in 96-well microplates and incubated for 6 min at room temperature. Then, 75 µL of sodium carbonate (Na_2_CO_3_) solution was added. After 2 h of incubation in the dark, the absorbance at 740 nm was read. The results were calculated using the gallic acid calibration curve (y = 7.9754x + 0.0187; *R*^2^ = 0.9938) and presented as mg gallic acid equivalents per gram of dry extract (mg GAE/g DE).

### 2.4. Evaluation of Total Flavonoid Content

The total flavonoid content was estimated as previously described by Chatatikun and Chiabchalard [19], with modifications suggested by Sembering et al. [20]. The ethanol extracts were mixed with 96% ethanol, 10% aluminum nitrate nonahydrate (Al(NO_3_)_3_ × 9H_2_O), and 1M potassium acetate (CH_3_COOK) in 96-microtiter plates and incubated for 40 min at room temperature. The absorbance was measured at 415 nm. The results were read from the quercetin calibration curve (y = 3.3703x + 0.2047; *R*^2^ = 0.9837) and are expressed as mg quercetin equivalents per gram of dry extract (mg QE/g DE).

### 2.5. LC-MS Analysis of Ethanol Extracts Obtained from Corn and Sunflower Crop Residues

The contents of phenolic components were determined using the LC-MS System 1260/6130 (Agilent Technologies, Waldbronn, Germany), which consisted of a quaternary pump, degasser, autosampler, column compartment, DAD, ESI ion source, and single quadrupole mass detector. Prior to analysis, the extracts were dissolved in ethanol to achieve a concentration of 10 mg/mL. The prepared solutions were filtered using membrane filters with a pore size of 0.45 µm. Separation was carried out on a reversed-phase Zorbax SB-Aq column (3 × 150 mm, particle diameter 3.5 µm, Agilent Technologies). The mobile phase consisted of 0.1% aqueous formic acid (A) and acetonitrile (B). The gradient elution protocol was as follows: 10–90% B (0–25 min), 90–90% B (25–27 min), and 90–10% B (27–30 min). The flow rate was set at 0.35 mL/min. UV spectra were measured in the range of 190–640 nm, while mass spectra were obtained in negative ion mode within the *m*/*z* range from 100 to 1000 using fragmentor voltages of 100 and 250 V. The spray chamber parameters included a gas temperature of 350 °C, drying gas flow of 10 L/min, nebulizer pressure of 40 psig, and capillary voltage of 3500 V. The extract constituents were identified by comparing their retention times, UV, and MS spectra with the corresponding parameters of the commercially available reference compounds obtained under the same chromatographic conditions. Quantification was performed using the external standard method, and the contents are expressed as mg of compound per 1 g of dry extract. Tricin was determined at 350 nm (y = 43243.30x + 51.60, *R*^2^ = 1, concentration range 0.0050–0.2500 mg/mL, LoD = 0.0017 mg/mL, LoQ = 0.0050 mg/mL), while chlorogenic acid (y = 3406.40x + 1.29, *R*^2^ = 0.9982, 0.0047–0.0280 mg/mL, LoD = 0.0015 mg/mL, LoQ = 0.0047 mg/mL), ferulic acid (y = 44074.88x + 6.94, *R*^2^ = 1, 0.0014–0.0700 mg/mL, LoD = 0.0003 mg/mL, LoQ = 0.0008 mg/mL), and *p*-coumaric acid (y = 98637.35x + 111.44, *R*^2^ = 0.9997, 0.0004–0.1200 mg/mL, LOD = 0.0023 mg/mL, LoQ = 0.0071 mg/mL) were quantified at 320 nm.

### 2.6. Evaluation of Antioxidant Activity

The free radical scavenging activity was evaluated using the protocol developed by Prieto [21] and Xiao et al. [22] for 1,1-diphenyl-2-picrylhydrazyl (DPPH) and 2,2′-azino-bis(3-ethylbenzothiazoline)-6-sulfonic acid (ABTS), respectively. For the DPPH assay, serial dilutions of the extracts (100 µL) were prepared in methanol in 96-microtiter plates. The obtained working solutions of the extracts were mixed with 0.2 mM DPPH solution and incubated in the dark at room temperature for 30 min. Subsequently, the absorbance at 517 nm was read in comparison to a blank containing methanol instead of the extract. The ABTS assay was also performed in 96-microtiter plates. The stock solution of ABTS free radicals was prepared 12–16 h before the experiment by mixing an equal volume of 7 mM ABTS and 2.45 mM potassium persulfate (K_2_S_2_O_8_) in an aqueous solution. After incubation in the dark, 4.3 mL was diluted to 100 mL in distilled water and the absorbance at 734 nm was adjusted to 0.74 ± 0.03. Serial dilutions of the extract were prepared in distilled water and mixed with 0.2 mL of ABTS working solution. The radical scavenging capacity (RSC) for both assays was calculated using the following equation:

RSC (%) = [(A_C_ − A_S_)/A_C_] × 100%

where A_C_ and A_S_ are the absorbance values of the blank and test solutions, respectively. The rates of the scavenged free radicals as a function of the extract concentration were used to generate a graph. The results were read from the obtained graph, expressed as IC_50_ values (mg/mL), representing the extract concentration that reduces free radicals by 50%, and compared with standard antioxidants (BHT and *L*-ascorbic acid).

The ferric reducing/antioxidant power assay (FRAP) was performed as described by Benzie and Devaki [23] with slight modifications. The aqueous working solutions were prepared from the extract and mixed with FRAP reagent (0.3 M Na-acetate buffer (pH 3.6): 10 mM TPTZ solution in 20 mM HCl: 20 mM FeCl_3_ in ratio 10:1:1 (*v*/*v*/*v*)) in a final volume of 0.2 mL. After incubation at 37 °C for 10 min, the absorbances were measured at 595 nm. The results were determined from the ferrous sulphate heptahydrate calibration curve and are expressed as µmol Fe^2+^ equivalents per mg of dry extract (µmol Fe^2+^ equivalents/mg DE).

### 2.7. Production Process of Pork Liver Pâtés

The commercial fresh shoulder lean pork meat, livers, and backfat used in the production of the pork liver pâtés were obtained from the local slaughter plant in Pećinci (Srem district, Serbia) at 24 h postmortem, all originating from Yorkshire × Landrace crossbreed pigs. The pork liver pâtés were produced in the experimental meat-processing facility at the Department of Food Hygiene and Technology, Faculty of Veterinary medicine, University of Belgrade. All pork liver pâtés were elaborated based on the same recipe similar to the commercial product [24], and the ingredients in the basic formulation per 1 kg of meat emulsion are presented in Table 1. In this study, four experimental groups of pork liver pâtés were prepared: a control batch (CON) of pork liver pâtés without added synthetic phenolic antioxidants or crop residue extracts; a batch formulated with the synthetic antioxidant butylated hydroxytoluene (BHT), added up to the highest concentration allowed by Serbian regulation [25] for this type of meat product (0.02%); a batch formulated with 1% of sunflower stalk residue extract (SSRE); and a batch formulated with 1% of maize stalk residue extract (MSRE). The selection of these extract concentrations was based on a preliminary study conducted in our laboratory (unpublished data), primarily considering the exhibited crop extracts’ antioxidant and antimicrobial activity and the sensory acceptability of the final product. For each formulation, 3.5 kg of raw material was used to produce the pork liver pâtés, the batches were made in triplicate, and these were elaborated independently in three production processes.

The pork backfat and livers were chopped into cubes approximately 1.5 cm^3^ in size, while the meat was processed in a mincer equipped with a plate featuring a hole with a 6 mm diameter. The fat was pre-cooked at 65 °C for 15 min. Initially, the livers and meat were comminuted in a cutter (Misowy 5.3l Sirman C6 Cutter, Polska) for 3 min. Subsequently, cubes of fat cooled at room temperature were added to the cutter along with the remaining ingredients and an appropriate amount of crop extract or synthetic antioxidant according to the formulation. All components were mixed in the cutter until a homogeneous raw batter was achieved. The meat batter was then vacuum-packed (100 g per bag, 35 bags per experimental group) and cooked by immersion in a hot water bath until it reached the meat core temperature of 72 °C for 15 min (aprox. 30 min). After the pâté samples were allowed to cool at room temperature, they were stored refrigerated at 4 ± 1 °C in the darkness for 90 days from the day of the manufacture (day 0). Units from each formulation of each replicate of pork liver pâtés were randomly taken at days 0, 20, 40, 60, and 90 after production for the following analysis: proximate chemical composition, fatty acid profile, lipid oxidative stability, physicochemical properties, instrumental color measurement, and microbiological analysis. A sensory analysis was carried out on day 30 of storage.

### 2.8. Determination of Pork Liver Pâtés’ Chemical Composition

The proximate chemical composition of the pork liver pâtés, including the contentsof fat [26], moisture [27], protein [28], ash [29], nitrite [30], and NaCl [31], was evaluated in triplicate according to official methods. All results are expressed as g/100 g of pork liver pâtés. The content of total phosphorus (natural and added) was estimated based on the official method [32] using photometric measurements conducted with a Halo DB-20/DB-20S (Dynamica, Livingston, UK). The energy value (Kcal/100 g pork liver pâté) was determined according to the Atwater coefficients corresponding to proteins (4.02 kcal/g), lipids (9.00 kcal/g), and carbohydrates (3.87 kcal/g).

### 2.9. Determination of Pork Liver Pâtés’ Physicochemical Properties

The pH of the pork liver pâtés was directly measured at room temperature (25 °C) using a pH meter (Testo 205, Testo AG, Lenzkirch, Germany) equipped with a digital identification system, temperature compensation sensor, and a proper glass electrode for penetration. The pH of the pork liver pâtés, three samples from each replicate of four groups, was measured three times by the same individual using the same pH meter consistently throughout the experiment. Before each series of measurements, the pH meter was calibrated at room temperature (25 °C) with pH 4.00 and 7.00 standard buffer solutions. Between each pH measurement, the pH meter electrode was rinsed immediately and thoroughly with distilled water and dried carefully with a clean paper towel.

The water activity (a_w_) of the pork liver pâtés was measured at room temperature (25 °C) using an a_w_-meter (FAst/1, GBX Scientific Instruments, Romans, France), following the guidelines in the instruction manual.

### 2.10. Determination of Pork Liver Pâtés’ Color

The instrumental color of the pork liver pâtés was automatically measured at room temperature (25 °C) after approximately 30 min of blooming time using a portable colorimeter (NR110, 3NH Technology Co., Ltd., Shenzhen, China) with the following settings: 8 mm aperture, 2° viewing angle, and D65 illuminant. Before measurement, the colorimeter was calibrated according to the manufacturer’s instructions. During the color measurement, the pork liver pâtés were placed on a white background, while the spectrally pure glass was put between the samples and the colorimeter. The color was measured on three pâté samples from each replicate of each formulation at three different randomly selected points. The results are expressed as lightness (L*), redness (a*), yellowness (b*), hue (h°), and chroma (C*) values. The numerical total color difference (ΔE) between the samples (S) was calculated with regard to the control group of pork liver pâtés (CON) on day 0 and day 90 based on Formula (1) and within the same pork liver pâté group between day 0 and day 90 (ΔE*) according to Formula (2) [33]:

(1) $$\Delta E=\sqrt{{\left(a_{s}-a_{c}\right)}^{2}+{\left(b_{s}-b_{c}\right)}^{2}+{\left(L_{s}-L_{c}\right)}^{2}}$$

(2) $$\Delta E^{*}=\sqrt{{\left(a_{90}^{*}-a_{0}^{*}\right)}^{2}+{\left(b_{90}^{*}-b_{0}^{*}\right)}^{2}+{\left(L_{90}^{*}-L_{0}^{*}\right)}^{2}}$$

### 2.11. Determination of Pork Liver Pâtés’ Fatty Acid Profile

The fatty acid profile of pork liver pâtés was determined following the methods previously described by Spirić et al. [34] and Glisic et al. [35]. Total lipids extraction from the pork liver pâtés was performed by the accelerated solvent extraction method with a hexane/isopropanol mixture. For fatty acid methyl esters (FAMEs) preparation, trimethylsulfonium hydroxide was used [36]. The separation and quantification of the FAMEs were performed using a gas chromatograph (Shimadzu 2010, Kyoto, Japan) equipped with a flame ionization detector (FID), split/split less injector, fused silica cyanopropyl HP-88 capillary column (100 m × 0.25 mm × 0.20 mm, J&W Scientific, Folsom, CA, USA), and workstation (Shimadzu GC Solution ver. 2.3). The characteristics and conditions of the separation of the FAMEs using gas chromatography were as follows: (i) the injector and detector temperatures were set at 250 °C and 280 °C, respectively; (ii) nitrogen was used as the carrier gas at a 1.33 mL/min flow rate and injector split ratio of 1:50; and (iii) the initial oven temperature of 125 °C increased at 10 °C/min to 175 °C and was held for 10 minutes, increased at 5 °C/min to 210 °C, held for 5 min, and then increased at 2 °C/min to a final temperature of 230 °C. Individual FAMEs were identified by comparing their retention times with a known standard FAME mixture. The determination of the fatty acid profiles was performed in triplicate at the beginning of refrigerated storage. Quantification was performed using heneicosanoic acid methyl ester (C21:0) as an internal standard. Data regarding fatty acid profiles are expressed as mg per 100 g total fatty acids. The proportions of polyunsaturated (PUFA), monounsaturated (MUFA), and saturated (SFA) fatty acid contents and the ratios of PUFA/SFA and n-6/n-3 were also calculated. The health lipid indices, atherogenic index (AI), thrombogenic index (TI), and the index of hypocholesterolemic/hypercholesterolemic fatty acids (HH), were computed as follows [37]:

AI = (C12:0 + 4 × C14:0 + C16:0)/(n-3 PUFA + n-6 PUFA + MUFA);

TI = (C14:0 + C16:0 + C18:0)/[0.5 × MUFA + 0.5 × n-6 PUFA + 3 × n-3 PUFA + (n-3 PUFA/n-6 PUFA)];

HH = (18:1n-9 + 18:2n-6 + 18:3n-3 + 20:4n-6 + 20:5n-3 + 22:6n-3)/(14:0 + 16:0)

### 2.12. Determination of Pork Liver Pâtés’ Lipid Oxidative Stability

The oxidative stability of the pork liver pâtés was determined in triplicate during the storage time based on the Thiobarbituric Acid Reactive Substances (TBARs) test using the combined method according to Tarladgis et al. [38] and Holland [39]. Briefly, 20 g of the pork liver pâtés was homogenized in distilled water and treated with HCl so a final pH of 1.5 was reached. The acidified homogenates were distilled using a distiller with a 50 mL bulb to collect the distillate. Afterwards, 5 mL of collected distillate and 5 mL of TBA reagent (0.3% 2-thiobarbituric acid in 90% acetic acid) were vortexed. After vortexing, the samples were heated in a constant-temperature boiling water bath at 95 °C for 35 min, and, thereafter, were cooled for 10 min at room temperature (25 °C). The reaction mixture absorbance was read at 532 nm using a Cecil CE 2021 spectrophotometer (Cecil Instruments Ltd., Cambridge, UK), and the calculated value is expressed as mg malonaldehyde per kilogram (mg MDA/kg) of pork liver pâté. The determination of the peroxide value (POV) was performed based on the ISO standard [40], as outlined in Glisic et al. [40], and the results are expressed as millimoles of active oxygen per kilogram of pork liver pâté (mmol O_2_/kg).

### 2.13. Microbiological Analysis of Pork Liver Pâtés

Microbiological analyses were performed in triplicate to evaluate the sanitary conditions of the pork liver pâtés from day 0 to day 90 of storage. On each examination day, 10 g from every pork liver pâté formulation was aseptically excised from the interior, transferred into sterile stomacher bags, homogenized with 90 mL of 0.1% of sterile peptone water, and blended in stomacher (Colworth 400, Seward, London, UK) for two minutes. Afterwards, appropriate serial decimal dilutions of each sample were prepared in 0.1% peptone water and inoculated onto the corresponding agar plates. The total viable count (TVC) and psychrotrophic bacteria count were determined on PCA by pour plates aerobic incubation at 30 °C for 72 h and at 7 °C for 10 days, respectively. *Enterobacteriaceae* microorganisms were isolated and counted on a VRBG agar by pour plates aerobic incubation at 37 °C for 24 h. The isolation and determination of lactic acid bacteria (LAB) were carried out on a MRS agar following incubation in anaerobic conditions at 30 °C for 72 h. The results are expressed as logarithms of colony-forming units per gram of pork liver pâté (log CFU/g).

### 2.14. Sensory Analysis of Pork Liver Pâtés

A sensory analysis of four pork liver pâté formulations was performed after 30 days of storage in three replicates by a trained panel with 7 experienced sensorists (4 females and 3 males, 35–55 years of age) selected from staff members of the Department of Food Hygiene and Technology (Faculty of Veterinary medicine, University of Belgrade). All panelists were regular consumers of pork liver pâté. Before testing, the panelists underwent a training session to define the attributes and descriptors to be evaluated in the pâtés. The test took place in the tasting room of the Department of Food Hygiene and Technology (Faculty of Veterinary Medicine, University of Belgrade), equipped with individual cabins. Rectangular pieces of about 1.5 cm × 2 cm were cut from the center of slices, coded with randomly selected 3-digit numbers, and served at room temperature in a randomized order to each panelist. Bread and tap water were also provided to the panelists to cleanse their palates between samples.

According to a quantitative descriptive analysis (QDA), the intensity of eight sensorial attributes was rated using a lineal structured scale from 0 (minimum intensity) to 7 (maximum intensity), with the ability to give semi-scores. The descriptors of the attributes were as follows: color (from light to dark); odor (from imperceptible to intense); cohesiveness (from low to high); homogeneity (from smooth to coarse); aroma (from imperceptible to intense); flavor, including saltiness, fattiness, and rancid flavor (from imperceptible to intense); adhesiveness (from low to high); and juiciness (from dry to juicy). At the end of the test, the panelists were asked to give a score from 0 to 7 for the products’ overall acceptability.

### 2.15. Statistical Analysis

The chemical composition and fatty acid profile were determined after the pâtés’ elaboration (day 0), and their sensory characteristics were evaluated after one month of storage, with three repetitions for each experimental replicate (4 treatments × 1 time period × 3 batches × 3 experimental replications). Throughout the storage period (0, 20, 40, 60, and 90 days), color, pH, a_w_, microbiology, POV, and TBARs were determined in three repetitions for each experimental replicate (4 treatments × 5 time periods × 3 batches × 3 experimental replications).

Statistical analyses were conducted with the SPSS software package version 20.0 (SPSS Inc., Chicago, IL, USA). Values from the crop residue extracts’ antioxidant capacity, proximate composition, fatty acid profile, and intensity of sensory attributes were evaluated using a General Linear Mixed Model (GLMM), with treatments as a fixed effect and the replicates as a random effect. For the pH, a_w_, microbiology, color, and lipid oxidation parameters’ data, the treatments and storage time were considered as fixed effects and the replicates as a random effect. No significant differences (*p* > 0.05) were observed in the variations among the replicates for any of the examined features. Except for the total phenol and flavonoid contents in the crop extracts, for which the independent samples *t*-test was used, the intergroup comparisons were appraised by one-way analysis of variance (ANOVA) followed by Tukey’s multiple comparison tests. Statistical significance was set at *p <* 0.05 or *p* < 0.01.

## 3. Results and Discussion

### 3.1. TPC, TFC, Antioxidant Activity, and Phenolic Compounds of Sunflower and Maize Stalk Residue Extracts

The total phenol and flavonoid contents in the maize stalk residue extract were higher than those in the sunflower stalk ethanolic extract (Table 2). However, this difference was significant only in the total phenolic compounds values (20.44 vs. 15.83 mg GAE/g dry basis; *p* < 0.05).

In previous studies, moderate to high TPCs [41], with values ranging from approximately 29 to about 390 mg GAE/g, have been observed in different plant extracts (grape seed, green tea leaves, chestnut leaves, beer residue, peanut skin, annatto seed flour, and persimmon flour) used as natural antioxidants in pork pâtés [9,42,43,44,45]. Contrastingly, Martín-Sánchez et al. [46] and Lorenzo et al. [47] formulated pork pâtés with date palm and seaweed extracts, which showed slightly lower but still moderate levels of total phenolic contents (14.5 and 12.8 mg GAE/g), consistent with the results of the present study.

The literary data on the contents of phenolic compounds in different parts of the sunflower and corn plants are limited [48,49,50,51]. Furthermore, to the best of our knowledge, there is no available information regarding the total phenol and flavonoid contents in sunflower crop residue, while the study by Vazquez-Olivo et al. [49] is one of the few where data on the phenolic compounds content in corn stover can be found.

Gai et al. [50] showed that the total phenolic content in the methanol extracts of sunflower aerial parts in the late stage of flowering was 21.7 mg GAE/g, a result comparable to the findings of the current study. Kamal [48] found a notably higher phenol and flavonoid content in sunflower methanol extracts, with the lowest average two-year values in stems (228.35 mg GAE/g and 55.33 mg QE/g, respectively), followed by roots, while the highest amount was reported in the leaves extract.

In the present study, the levels of *p*-coumaric acid, ferulic acid, and tricin in the ethanol extract of maize crop residues were determined using LC-DAD-ESI-MS and found to be 1.64 mg/g, 1.21 mg/g, and 1.99 mg/g, respectively. Additionally, chlorogenic acid was detected in the ethanol extract of sunflower field waste at a concentration of 2.40 mg/g. Previous studies have revealed that chlorogenic acid is the most prevalent phenolic acid in extracts of sunflower kernels, shells, sunflower seed flour, and seed cake [52,53,54]. However, in the late flowering sunflower plant extract, the predominant compound was 3,5-di-*O*-caffeoylquinic acid (12.82 mg/g), followed by chlorogenic acid (7.04 mg/g) [50]. Considering that the efficiency of extraction is influenced by the type of solvents and the composition of the raw material, previous studies have demonstrated that an ethanol solution is suitable for extracting phenolic compounds from the sunflower matrix [54,55]. Alexandrino et al. [54] further indicated that ethanol extracts of sunflower flour also contain other soluble compounds, such as sugars and proteins. Corroborating the literature data presented, in the present study, chlorogenic acid was identified in the investigated sunflower extract, however, an approximately three times lower content in our sample was observed than that previously reported [50]. Furthermore, a compound with the molecular ion at *m*/*z* 515 was detected and a retention time corresponding to that of the reference standard of 3,5-di-*O*-caffeoylquinic acid. Nevertheless, we were unable to confirm its identity with certainty, due to low-quality UV spectra resulting from its very low concentration.

Previously, Vazquez-Olivo et al. [49], by evaluating the distribution of the phenolic compounds in different parts of maize, showed that TPC in maize stem extract was 9.34 mg GAE/g DW, indicating the significant effect of plant organ on TPC, as well as on the bound and free phenolic amounts. In accordance with these results, Adekunle et al. [51] also found a lower TPC in the ethanol extract of corn stalk (just below 2 mg GAE/g) compared to the results of our study. On the contrary, in Vijayalaxmi et al.’s [10] study, higher total polyphenols and total flavonoids contents were found (426.5 mg GAE/g and 87.2 mg QE/g, respectively) in a corn husk extract obtained using 50% ethanol. Further, by examining different agro-industrial byproducts, Abbasi-Parizad et al. [56] determined a TPC of 12 mg GAE/g DW in the aqueous ethanolic extract of red corn cob, which is also lower compared to the result obtained for our maize stalk residues. These variations could be explained by the fact that different organs are exposed to environmental stress differently, among which corn husks are the most exposed, followed by the cob, stalk, or grain [49]. In Abbasi-Parizad et al.’s [56] study, phenolic acids constituted 28.9% of the total phenols, whereas flavonoids accounted for the remaining 71.1%. The most prevalent phenolic acids were chlorogenic acid and ferulic acid, while catechin and epicatechin were the predominant ones among flavonoids. Galeana-López et al. [57] discovered that the most abundant phenolic compound in corn husk ethanolic extract was ferulic acid (12.93 mg/g), followed by *p*-coumaric acid (5.74 mg/g). In our sample, the content of *p*-coumaric acid was higher than that of ferulic acid, and both acids were present in lower amounts compared to the mentioned study. Additionally, we showed that the flavon tricin occurred in an appreciable quantity. In contrast to the findings reported by Abbasi-Parizad et al. [56], we did not detect chlorogenic acid, catechin, or epicatechin in the examined maize extract.

As can be seen in Table 3, the determined in vitro antioxidant activity was related to the concentration of the phenolic compounds of the crop residue ethanolic extracts. Namely, the radical scavenging capacity evaluated through DPPH and ABTS assays was more than 2-fold greater for maize stalk residue extract (IC_50_ were 0.41 and 1.72 mg/mL, in DPPH and ABTS tests, *p* < 0.05, respectively) than that for sunflower stalk residue extract. Further, the ferric ions reducing power of MSRE was also significantly higher compared to that of SSRE (0.25 and 0.14 µmol Fe^2+^/g, *p* < 0.05, respectively). In comparison with standard compounds, the synthetic antioxidants BHT and *L*-ascorbic acid, significantly higher concentrations of MSRE and SSRE were required to achieve a 50% decrease in the initial DPPH and ABTS levels (*p* < 0.05). Further, the FRAP values for both extracts were significantly lower (*p* < 0.05) than those of the positive control. In contrast to the present results, the 50% methanolic extract of corn husk showed a higher reducing power and better DPPH scavenging activity (IC_50_ 0.024 mg/mL) compared to the positive control ascorbic acid (0.038 mg/mL) [10].

Gai et al. [50] demonstrated that the antioxidant scavenging activity of sunflower extract changes during different growth stages, with the late flowering plant extract showing results of 0.18 mg/mL and 0.34 µmol Fe^2+^/mg for DPPH and FRAP, respectively. Despite lower antioxidant activity, our results are consistent with a positive correlation between the TPC and antioxidant activity of the sunflower extract presented in this study. However, Abbasi-Parizad et al. [56] found that the total antioxidant activity did not align with the trend of TPC across various agro-industrial byproduct extracts, including red corn cobs. Vazquez-Olivo et al. [49] reported variations in the antioxidant capacity among different maize organs. For example, the highest antioxidant capacity for DPPH was found in the free phenolic extracts from the stalk, while the highest values for the bound phenolic extract were identified in the cob and husk.

The compounds quantified in the examined extracts are known to display antioxidant effects in experimental settings [58,59,60]. Therefore, it can be concluded that the total antioxidant activity arises, at least in part, from their presence.

Finally, the differences in the antioxidant activity of the sunflower and corn extracts observed in the mentioned studies were due to their different phytochemical profiles, which depends on numerous factors, including the environmental, agronomic, and meteorological conditions and the extraction procedure (solvent polarity, temperature, and extraction time). Additionally, these differences also result from the methods used for the evaluation of antioxidant activity, since they are based on different molecular mechanisms such as electron transfer and hydrogen donation [10,49,57].

### 3.2. Proximate Composition of Pork Liver Pâtés

The proximate compositions and energy values of the control pork liver pâtés and pâtés made with BHT, sunflower, and maize residue extracts are given in Table 4. No significant differences (*p* > 0.05) were found among the different formulations regarding moisture, protein, fat, ash, NaCl, nitrite, and P level (expressed as P_2_O_5_), as well as energy value. Given the same raw material composition across all batches, these results were anticipated. Namely, all the formulations were in the accordance with the Serbian legislation, which defines a minimum 9% protein content in liver pâtés and sausages [61]. The present results corroborate previous studies that have evaluated similar liver pâté formulations, where the addition of plant extracts and essential oils as natural antioxidants did not affect the chemical composition of the meat products [6,44,62].

### 3.3. Fatty Acid Composition of Pork Liver Pâtés

The fatty acid (FA) composition and key nutritional indices of both the control and pork liver pâtés prepared with crop residue extracts at the beginning of refrigerated storage are presented in Table 5. The main FA fraction in all the pâté formulations was composed of MUFAs (ranging from 44,880–45,086 mg/100 g), followed by SFAs and PUFAs. The primary fatty acids determined were C18:1 > C16:0 > C18:0 > C18:2, with a significantly higher content of linoleic acid in the SSRE and MSRE pâtés, consequently leading to an increased n-6 content in these pâtés (*p* < 0.01). These results are consistent with those reported by other authors [42,45,63], where pork liver pâtés were primarily composed of MUFAs, with oleic acid identified as the predominant fatty acid. Further, corroborating the present findings, the incorporation of natural extracts from seaweed or various agro-industrial residues (beer residue, chestnut leaves, and peanut skin) did not affect or lead to minimal deviations in the FA content of the pork liver pâtés during processing [44,64]. However, unlike our results, Munekata et al. [62] did not find differences in the n-6 and total FA contents between the negative and positive controls (BHT) of ground sheep patties, as well as patties with an added 1% peanut skin extract, both at the beginning and after a 20-day storage period.

The addition of sunflower and maize crop residue extracts significantly increased the PUFA content and PUFA/SFA ratio of the pork liver pâtés (*p* < 0.01); however, this ratio was lower than the optimal recommended values (0.5–0.7) in the Mediterranean diet [65]. In contrast to these results, the addition of green tea extract slightly decreased the PUFA/SFA ratio in liver pâtés [42]. The n-6/n-3 ratios in our pâté formulations (around 25) exceeded those reported in other pork liver pâtés (from 7 to 18.69) [42,63,66,67] and were several times higher than the nutritionally recommended limit of 4 [68].

By calculating the health lipid indices, it can be noticed that, despite a significantly higher n-6 content in the pâtés with added crop residue extracts, this increase was not sufficient to reflect a difference in the AI. The absence of differences in the n-3 content between different pâtés batches resulted in an expected TI value of around 1.5 in all formulations. Meat products with a lower AI and TI and higher HH values are characterized by a healthier lipid composition, with a potentially favorable effect on the cardiovascular system [69]. Following the recommended levels for AI and TI, which should be below 1 [69], our pâtés, featuring a fat content of around 28%, typical for commercial meat products incorporating pork liver [63], met this criterion exclusively for AI, while HH was approximately 1.9.

### 3.4. Water Activity (a_w_) Values of Pork Liver Pâtés

The water activity was influenced by the inclusion of different extracts and storage times, with the interaction between both factors also proving to be significant (Table 6, *p* < 0.01). During the first 20 days of storage, the a_w_ values did not differ between pâté formulations, except for a significantly lower a_w_ value in the pâtés with sunflower extract added on day 0 (0.941). The crop residue extracts and BHT led to an increase in the a_w_ values on days 40 and 90 (*p* < 0.01), while, on day 60, only the pâtés containing maize crop residue and BHT demonstrated higher a_w_ values compared to the control (*p* < 0.01). The water activity of each pâté formulation showed a tendency to increase from the beginning of the experiment, starting at approximately 0.945, until day 40 of storage. However, after two months of storage, a continuous decline in a_w_ values was observed, reaching approximately 0.907 by the day 90. Comparable results were reported by Martín-Sánchez et al. [46], who reported that the addition of date palm paste, either alone or in combination with annatto extract, exhibited an increasing trend in a_w_ values during the initial storage period across all formulations of spreadable pâtés, with a noticeable decline observed after 3 weeks of storage. Furthermore, Amaral et al. [70] found a similar behavior in reducing the a_w_ values of lamb pâtés packaged in a polyamide casing system during a 90-day storage period. Contrastingly, other authors reported minimal or no alterations in a_w_ values in liver pâtés with natural ingredients added [8,45].

### 3.5. pH of Pork Liver Pâtés

Table 7 displays the evolution of the pH values in the pork liver pâtés stored under refrigeration at 4 °C. The pH was significantly affected by the formulation, storage time, and interaction of these factors (*p* < 0.01). The inclusion of crop extracts resulted in a significant decrease in pH compared to both the control and BHT pâtés (*p* < 0.01) over the 90-day storage period. Despite these statistically significant differences, variations in this parameter were small, ranging from 6.33 to 6.52, which is considered to be usual for this kind of product [71]. This aligns with the findings of Martín-Sánchez et al. [46] and Lorenzo et al. [47], who reported the addition of grape seed extract, green tea extract, and date palm coproducts paste, rich in organic acids, to decrease the pH value in raw minced porcine patties and spreadable pork liver pâtés. Similar patterns in pH evolution within each batch during the 24-week storage period were observed by Pateiro et al. [42] in pig liver pâtés with 0.1% of tea, chestnut, and grape seed extracts. Additionally, Pateiro et al. [9] showed a dose-dependent pH reduction in pig liver pâtés with increasing concentrations of grape and tea extracts. However, significant modifications in the pH of pork liver pâtés elaborated with different natural extracts from seaweed, beer residue, and chestnut leaves were not found, indicating that predominant active compounds in these extracts, mainly phenolic acids, did not affect the pH of the pâté formulations [44,64].

### 3.6. Color of Pork Liver Pâtés

The color characteristics of the pâtés were affected by incorporating crop residue extracts (Table 7). Initially, pâtés with sunflower crop extract were significantly darker (L* = 50.92), whereas maize crop extract reduced the a* value and increased the b* and hue values of the pâtés (*p* < 0.01). The pâtés with added BHT were significantly redder (a* = 13.24), with a higher chroma value (C* = 18.26) compared to the control (C* = 17.39) (*p* < 0.01). These differences between formulations persisted after 20 days of storage (*p* < 0.01). However, after two and three months of storage, both formulations with added extracts were significantly darker compared to the control pâtés (*p* < 0.01). At the end of storage, the addition of sunflower crop extract significantly increased the a*, b*, and chroma values and decreased the hue value of the pâtés, while the maize extract significantly increased the proportion of yellow (b*) and decreased the proportion of red color (a*) in the pâtés (*p* < 0.01). Following these findings, Pateiro et al. [9] also showed that the color of tea and grape seed extracts incorporated into the pig liver pâtés led to the different color modifications.

Color modification due to the addition of BHT and crop residue extracts could be regarded as observable, since the total color difference (ΔE) compared to the control pâtés on days 0 and 90 was greater than 2 [72], with the most pronounced changes observed in the SSRE pâtés (5.16 and 8.33, respectively). During storage, color changes in the pâtés were also observed, with the highest values of the total color difference (ΔE*) from 0 to 90 days being found in the control (3.35) pâtés and pâtés with sunflower crop extract added (3.15).

While no changes in a* and b* values were observed in the control pâtés during storage, these pâtés were significantly lighter after two months of storage (*p* < 0.01). On the other hand, the evolution of L* values during storage in formulations with added BHT or crop extracts was somewhat inconsistent. The a* and chroma values significantly increased after 2 and 3 months of storage compared to day 0 in formulations with added crop extracts (*p* < 0.01), while the b* value significantly increased on day 90 of the study (SSRE and MSRE, 14.34 and 15.87, respectively), with a variable trend in this parameter during storage. In contrast to these results, Pateiro et al. [9] demonstrated that, with increasing amounts of natural antioxidants (grape and tea extracts), both the a* and b* values of the pâtés were lower.

Initial differences in the lightness (L*) and yellowness (b*) of the SSRE and MSRE pâtés in comparison with the control pâtés could be attributed to the incorporation of 1% of brownish-yellow components of the sunflower and maize crop extracts and the operational process. The mentioned differences were even more pronounced after three months of storage, and the changes were particularly evident in the pâtés with sunflower crop extract, which also exhibited the lowest oxidative stability. Specifically, previous studies have reported that the oxidation and oxygenation of myoglobin during the preparation and storage of cooked meat products lead to color changes, primarily, an increase in the b* value and the development of a brownish-grey color [5,73]. However, the highest total color change value after the storage of the control pâtés suggests that, in addition to oxidative processes, other factors such as chemical composition, structural modifications of the meat emulsion, and heat treatment could also be responsible for changes in pâté color parameters [73,74]. These results are consistent with previous studies that have investigated the impact of storage and the addition of plant antioxidants on the color and oxidation parameters in various pâté formulations [5,43,70,74].

### 3.7. TBARs and Peroxide Value (POV)

Significant effects of treatment, storage, and their interaction on lipid oxidation parameters were observed. Therefore, the results, categorized by groups of pâtés and different examination days, are presented in Table 8.

A significantly higher peroxide value was observed in the SSRE and MSRE pâtés compared to the control and BHT pâtés (*p* < 0.01), continuously increasing in all formulations from day 0 to day 60 of storage. On the last day of storage, a decrease in POV was recorded, where the difference between the control and pâtés with maize crop residue extract (1.275 and 1.195 mmol/kg, respectively, *p* > 0.05) was not determined, while the SSRE pâtés had the highest POV (2.515 mmol/kg). Corroborating our results, Pateiro et al. [42] showed that BHT most effectively prevented the formation of primary lipid oxidation products that occur during the elaboration and thermal treatment of the pâtés. In contrast, after 24 weeks of storage, the same authors observed more than a 2.5 times lower peroxide value in pâtés enriched with chestnut and grape seed extracts than that in the control group. Likewise, Van Cuong and Chin [43] found that an increasing concentration of annatto seed powder (from 0.1 to 0.5%) resulted in a reduced peroxide value in pork patties throughout storage. Limited research has been conducted on the analysis of peroxide value in liver pâtés, and its validity as an indicator of lipid oxidation is questionable due to the low stability of peroxides, which readily break down into non-peroxide compounds [43]. According to the Codex Alimentarius, the peroxide value of animal fats intended for consumption should be below 10 mEq of active oxygen/kg [75].

During refrigerated storage, an increasing trend in TBARs values was evident across all pâté formulations. In the initial two months, the pâtés elaborated with crop residue extracts exhibited significantly higher TBARs values, with the SSRE group had the highest TBARs (ranging from 0.195 to 0.275 mg MDA/kg). After 90 days of storage, the BHT pâtés showed the lowest TBARs value (0.195 mg MDA/kg), and no significant differences were observed between the control and MSRE pâtés (*p* > 0.05). Meanwhile, the SSRE pâtés maintained the highest value at 0.450 mg MDA/kg.

Based on the in vitro tests of the present study and previously published findings on the same extracts [17], it appears that the ethanolic sunflower and maize residue extracts could exhibit antioxidant and antimicrobial activity. Namely, it was expected that the addition of sunflower and maize stalk residue extracts, considering that these extracts contain moderate TPC levels [41], would reduce lipid oxidation in the pork liver pâtés. However, throughout the storage period, the lowest TBARs value was observed in the pâtés with added BHT, followed by the control pâtés. In other words, the results suggest the opposite—the ethanol extracts used not only failed to provide a sufficient protective effect, but also induced a pro-oxidative effect in the pork liver pâtés. Since there are no available literature data regarding the incorporation of sunflower and maize residue extracts into food matrices, it is challenging to define all factors that could contribute to such an effect.

Many types of plant extracts used as natural antioxidants in pâtés have demonstrated substantial antioxidant activity in both, in vitro tests and in preventing lipid oxidation in these diverse meat products during cold storage [9,42,43,47,64,76]. However, Munekata et al. [44] noted no impact of agro-industrial by-product extracts on TBARs’ generation. Moreover, several studies have reported increased lipid oxidation in pork liver pâtés containing extracts rich in phenolic compounds. Specifically, the inclusion of date palm co-product paste in quantities of 2.5% and 7.5% heightened the lipid oxidation in spreadable pork liver pâtés [46]. Chestnut aqueous extract compromised the lipid stability of modified atmosphere-packaged pork patties [47]. Lucas-González et al. [3] observed significantly higher TBARs values in pork liver pâtés enriched with persimmon flour. The pro-oxidative effect of mushroom flours in liver pâtés was also determined by Cerón-Guevara et al. [77]. Furthermore, it has been shown that higher concentrations of rosemary essential oil (600 ppm) lead to increased oxidative deterioration in frankfurters made with meat from intensively reared white pigs [78].

The obtained results can be partially attributed to the likelihood that the antioxidant–pro-oxidant outcomes of crop residue extracts are influenced by the chemical characteristics of the ingredients in the complex emulsified biphasic system of the pork pâté and the overall manufacturing process [46,79]. Indeed, the interaction between extracts’ compounds, mainly phenolics, and other active substances present in the pâté matrix, such as sugars, nitric oxide, and Fe^3+^, could promote lipid peroxidation [3,80]. Furthermore, besides the fact that sunflower and maize stalk residue extracts had a low miscibility in the pâtés’ emulsion, the use of a cutter during ingredient comminution and mixture modifies the lipid state and iron availability in the pâtés, thereby also influencing the efficiency of the phenolic compounds in the extracts [44,46]. Additionally, the thermal treatment applied during pâté production may lead to the degradation of phenolic compounds in crop extracts, reducing their concentration and antioxidant potential [76]. Concurrently, heat releases pro-oxidative agents such as iron from myoglobin, which catalyzes lipid oxidation and leads to the formation of MDA from polyunsaturated fatty acids [77].

Our study’s results affirm earlier assertions that 0.009% nitrites are effective in preserving low TBARs values [79], as evidenced by all the pâté formulations, including the control, exhibiting TBARs values below the critical threshold of 1 mg MDA/kg, indicating the chemical deterioration of the product [43]. Furthermore, in the present study, as all formulations contained added nitrites, data related to the independent effect of extracts in meat systems are not available. Therefore, the antioxidative effect must be considered through the interaction of nitrites and crop residue extracts. One of the primary mechanisms through which nitrites exert an antioxidative effect in meat products is by binding NO to myoglobin, forming nitrosylmyoglobin, thus preventing the release and oxidation of Fe^2+^ [81]. In a study of the interaction of nitrites with green tea extract in pepperoni, Lin et al. [79] proposed that, given NO’s potential roles as a reducing, oxidizing, and nitrosating agent, there is a likelihood of its interaction with reductants, such as our crop residue ethanolic extracts. Moreover, various in vitro studies have documented the capability of plant extracts to scavenge or inhibit the production of peroxynitrite and NO [82,83]. Although there is still insufficient evidence regarding how the combination of plant extracts and nitrites may either reduce or potentially enhance lipid oxidation, in agreement with our results, similar effects have been observed in other meat products, such as Milano salami and emulsion-type pork sausage [84,85]. The interaction between crop residue extract and nitrite could also contribute to alterations in the fatty acid composition of pâtés, particularly the significantly higher levels of n-6 and PUFAs in the SSRE and MSRE pâtés, potentially accelerating the lipid oxidation in these formulations during storage [79].

Finally, aligned with our microbiological analysis results showing significantly higher microbial counts in the control and BHT pâté formulations compared to those enriched with the sunflower and maize crop residue extracts, one might infer that the degradation of lipid oxidation products occurred due to the presence of microbial catalysts, resulting in a reduced TBARs value in the pâtés without extracts added [86].

### 3.8. Microbiological Analyses

The microbial count was influenced by both the formulation of the pâtés and the storage time, with a significant interaction observed between these factors (*p* < 0.01) (Table 9). During the storage period, analyses revealed that *Enterobacteriaceae* were not detected in any of the pâté samples, confirming the results from previous studies [46,74], where it was suggested that the absence of these bacteria results from effective thermal treatment and good storage conditions, preventing post-processing microbial contamination.

The initial TVC, LAB, and psychrotrophic bacteria counts were < 2 log CFU/g in all formulations, with a significant decrease in the pâtés elaborated with crop residue extracts compared to the control and BHT pâtés (*p* < 0.01). Consistent with findings reported by Van Cuong and Chin [43], the microbial count increased with an increasing storage time. The number of aerobic bacteria and LAB remained similar throughout storage, reaching values above 5 log CFU/g only in the control and BHT pâtés for TVC, while the LAB count was below 5 log CFU/g in all pâté formulations. At the end of the storage period, a slightly lower psychrotrophic bacteria count was found, ranging from 4 to 4.6 log CFU/g for the SSRE and control pâtés, respectively.

Similar findings were observed in the study by Munekata et al. [62], where peanut skin extract did not impact the initial TVC in pâtés. However, over the storage period, formulations with BHT and peanut extracts exhibited lower TVC and LAB counts compared to the control. Additionally, high concentrations of date paste, either alone or combined with annatto extract, have been demonstrated to effectively inhibit microbial growth in pork liver pâtés [46]. Consistent with our results, patties with added BHT, green tea, and grape seed extracts displayed reduced TVC, LAB, and psychotropic aerobic bacterial counts relative to the control [47].

In a prior study, Glišić et al. [17] demonstrated the antimicrobial potential of sunflower and maize stalk residue ethanolic extracts, attributing it to the presence of phenolic bioactive compounds. The minimal inhibitory concentration (MIC) for these extracts was found to be 320 μg/mL for *Salmonella* Typhimurium, *Salmonella* Enteritidis, *Staphylococcus aureus*, *Escherichia coli*, *Listeria monocytogenes*, and *Yersinia enterocolitica*. Similarly, Alexandrino et al. [54] reported the antibacterial activity of the ethanolic extract of sunflower flour against certain Gram-positive and Gram-negative bacteria, primarily ascribing this effect to the presence of chlorogenic acid in the extract. Specifically, chlorogenic acid has the ability to bind to bacterial membranes, causing damage to the membrane integrity, potentially leading to bacterial death [87]. Conversely, some studies have found that various plant extracts, such as seaweed and annatto seed, did not impact the TVC and LAB in pork liver pâtés during storage [43,64]. In contrast, Cerón-Guevara et al. [77] observed that the inclusion of mushroom flours in liver pâtés led to an elevated total microbial count (>5 log CFU/g) due to the contamination of spore-forming bacteria by mushroom flours that survived thermal treatment.

The phenolics identified in the examined extract have scientifically confirmed activity against a wide range of microorganisms, such as Gram-positive and Gram-negative bacteria [54,58,59,60,87]. Hence, it can be expected that they contributed to the observed activity.

It can be inferred that the microbial growth in all our pâté formulations did not surpass the critical value for this type of product (6 log CFU/g), suggesting the absence of product spoilage after three months of storage [88]. This outcome is likely attributed to the synergistic effects of heat treatment, the incorporation of additives such as sodium chloride and nitrite, and the adherence to hygienic processing conditions and refrigerated storage [70]. Moreover, the microbiological quality of the pork liver pâtés was enhanced by the addition of the sunflower and maize stalk residue extracts.

### 3.9. Sensory Analyses

In previous studies addressing the incorporation of plant extracts into matrices of diverse types of liver pâté, the primary focus was on monitoring physicochemical characteristics and oxidative stability. The evaluation of product appearance predominantly relied on instrumental color measurement [5,9,42,43,44,46,47,64]. Given that the success of introducing new ingredients into meat and meat products is substantially dependent on consumer acceptability, gauged by the deviation in main sensory attributes compared to conventional products [89], there are limited studies that have conducted sensory evaluations of pâtés with added plant extracts as natural antioxidants [3,8,62,77,90].

**Figure 1:**
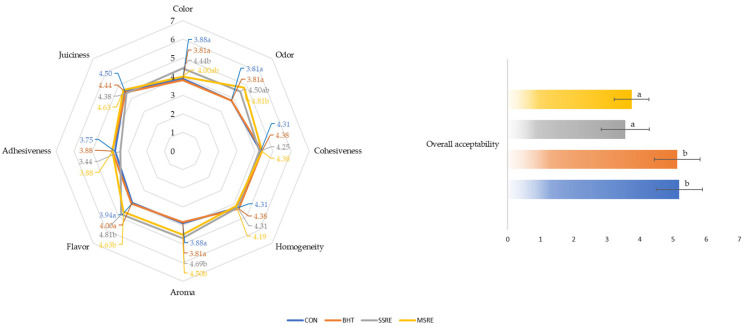
Intensity of the sensory attributes of pork liver pâtés and scores for overall acceptability. CON—control pâtés; BHT—pâtés with butylated hydroxytoluene added; SSRE—pâtés with sunflower stalk ethanol extract added; and MSRE—pâtés with maize stalk ethanol extract added. ^a,b^ Different lower-case superscript letters for each attribute indicate a significant difference between groups (*p <* 0.05); n = 3 for each group of pork liver pâtés in triplicate.

The findings from the sensory analyses of four different pâté formulations are synthesized in Figure 1. Trained panelists noted significant impact on color, odor, aroma, and flavor due to the addition of crop residue extracts (*p* < 0.05). Specifically, regarding color, the SSRE pâtés were assessed as being significantly darker compared to both, the control and BHT pâtés (*p* < 0.05). This observation aligns with objective color measurements, validating that the inclusion of sunflower stalk residue extract, due to the extract’s coloration, increased the redness (a*) and reduced the lightness (L*) of the pâtés. Comparable color alterations were documented by other researchers [3,43] when incorporating annatto seed and persimmon powders, underscoring color intensity as the most affected sensory attribute. Given the paramount importance of color in meat products, such deviations can significantly impact the overall acceptability of pâtés among consumers [91].

It was also shown that the formulations with the addition of crop residue extracts had a significantly more intense flavor and aroma (*p* < 0.01), while heightened odor intensity was only observed in the MSRE pâtés (*p* < 0.01). These sensory characteristics scores could be related to the lipid oxidation results and the presence of fat-oxidized metabolites. Namely, the higher POV and TBARs values in the SSRE and MSRE formulations could be responsible for the poor flavor ratings of these pâtés [43,71]. Other authors have also linked lipid oxidation to the presence of off-odor and off-flavor in patties, however, unlike our results, in their studies, natural plant extracts were effective in reducing rancid odor and flavor development [62,90].

The inclusion of crop ethanol extracts had no adverse impact on other sensory attributes, such as the cohesiveness, homogeneity, adhesiveness, and juiciness of the pâtés (*p* > 0.05), indicating the successful incorporation of the residue extract paste into the pâtés’ emulsion. Nevertheless, despite these aspects, the observed alterations in color, aroma, and flavor resulted in lower overall acceptance scores for the pâtés with crop residue extracts compared to the control and BHT formulations. Notably, the SSRE pâtés received the lowest ratings, approaching the acceptability threshold at 3.56. Earlier studies have indicated that the incorporation of plant extracts, while impacting color, did not yield significant differences in other sensory attributes. In fact, overall preferences, in some instances, were rated even more favorably than control pâté formulations [3,8]. The challenges arising from the introduction of new ingredients with potential negative effects on the sensory quality of pork liver pâtés can be effectively addressed by employing additives and seasonings. These components play a crucial role in controlling the color and modulating the intense flavor and aroma of pâtés, ultimately enhancing consumer acceptability [43,77].

## 4. Conclusions

The in vitro tests demonstrated that the ethanolic extracts from sunflower and maize crop residues exhibited antioxidant capacity to some extent; nevertheless, this was significantly lower than synthetic antioxidants. When incorporated into pork liver pâtés, these extracts exhibited a noticeable antimicrobial effect; however, they proved ineffective in preventing lipid oxidation. Specifically, the crop residue extracts displayed a pro-oxidant effect when compared to the use of only nitrites and a nitrites/BHT combination in the pork liver pâtés during storage. The addition of crop residue extracts influenced the instrumental color and intensified the flavor and aroma of the pork liver pâtés, resulting in lower acceptability scores for the product. The work is still in progress to completely elucidate the phenolic profiles of sunflower and maize stalk residue extracts and whether their composition could be influenced by the modification of processing or extraction to increase the amount of antioxidant compounds. To validate the potential of these agricultural residues as a source of natural preservatives for meat product applications, forthcoming studies should explore the mechanisms of interaction between the extract compounds and the components of pork liver pâtés. Additionally, investigations into the structural and functional changes that occur during processing and conservation treatments are essential for understanding the observed pro-oxidative effect and its detrimental impact on sensory characteristics.

## Figures and Tables

**Table 1:** Liver pâté formulations (g/kg).

Ingredients	Batches			
	CON	BHT	SSRE	MSRE
Liver	330	330	330	330
Subcutaneous fat	300	300	300	300
Lean meat	207	207	207	207
Water	125	125	125	125
Soy protein isolate	15	15	15	15
Curing salt (0.6% nitrite in NaCl)	15	15	15	15
Sodium tripolyphosphate	5	5	5	5
Mix of spices	3	3	3	3
BHT	-	0.2	-	-
Sunflower stalk residues extract	-	-	10	-
Maize stalk residues extract	-	-	-	10

CON—control pâtés; BHT—pâtés with butylated hydroxytoluene added; SSRE—pâtés with sunflower stalk ethanol extract added; MSRE—pâtés with maize stalk ethanol extract added; the concentrations of BHT and crop residue extracts were calculated on the basis of total ingredients.

**Table 2:** The total phenol and flavonoid contents in the ethanol extracts from sunflower and maize waste material (mean ± SD).

	SSRE	MSRE
TPC (mg GAE/g) ^1^	15.83 ± 0.30 ^a^	20.44 ± 0.23 ^b^
TFC (mg QE/g) ^2^	8.98 ± 0.34	9.37 ± 0.83

TPC—total phenol content; TFC—total flavonoid content; SSRE—sunflower stalk ethanol extract; and MSRE—maize stalk ethanol extract. The results were obtained from three different experiments performed in triplicates; ^a,b^ Different superscripts within the same row indicate a significant difference (*p* < 0.05). ^1^ mg of gallic acid equivalents per g of dry extract. ^2^ mg of quercetin equivalents per g of dry extract.

**Table 3:** Antioxidant activity of the ethanol extracts from sunflower and maize waste material (mean ± SD).

Tests	Extract/Positive Control			
	SSRE	MSRE	BHT	*L*-AA
DPPH IC_50_ (mg/mL)	1.02 ± 0.01 ^a^	0.41 ± 0.02 ^b^	0.038 ± 0.001 ^c^	0.0057 ± 0.0003 ^d^
ABTS IC_50_ (mg/mL)	4.53 ± 1.20 ^a^	1.72 ± 0.10 ^b^	0.04 ± 0.002 ^c^	0.0316 ± 0.0003 ^d^
FRAP (µmol Fe^2+^/g)	0.14 ± 0.01 ^a^	0.25 ± 0.01 ^b^	0.41 ± 0.03 ^c^	1.618 ± 0.004 ^d^

SSRE—sunflower stalk ethanol extract; MSRE—maize stalk ethanol extract; BHT—butylated hydroxytoluene; and *L*-AA—*L*-ascorbic acid. The results were obtained from three different experiments performed in triplicates; ^a,b,c,d^ Different superscripts within the same row indicate a significant difference (*p* < 0.05).

**Table 4:** Proximate composition and energy value of four pork liver pâté formulations (mean ± SD).

Item	Batches				*p* Value
	CON	BHT	SSRE	MSRE	
Moisture (%)	54.77 ± 0.31	54.67 ± 0.24	54.50 ± 0.71	54.44 ± 0.69	0.6875
Protein (%)	14.39 ± 0.04	14.50 ± 0.17	14.40 ± 0.14	14.42 ± 0.12	0.3777
Fat (%)	27.95 ± 0.31	27.97 ± 0.21	28.32 ± 0.53	28.29 ± 0.71	0.3941
Ash (%)	2.86 ± 0.02	2.81 ± 0.01	2.86 ± 0.01	2.83 ± 0.07	0.0970
NaCl (g/100 g)	1.75 ± 0.13	1.61 ± 0.02	1.59 ± 0.08	1.78 ± 0.19	0.0564
NaNO_2_ (mg/kg)	26.11 ± 2.92	27.64 ± 1.94	29.35 ± 1.48	27.40 ± 1.95	0.1057
P_2_O_5_ (g/kg)	6.70 ± 0.16	6.64 ± 0.11	6.69 ± 0.03	6.76 ± 0.05	0.2727
Energy value (Kcal/100 g)	309.10 ± 2.75	309.70 ± 1.94	312.50 ± 5.16	312.30 ± 6.26	0.4455

CON—control pâtés; BHT—pâtés with butylated hydroxytoluene added; SSRE—pâtés with sunflower stalk ethanol extract added; MSRE—pâtés with maize stalk ethanol extract added; n = 3 for each group of pork liver pâtés in triplicate.

**Table 5:** Fatty acid composition (mg per 100 g total fatty acids) of the pâté formulations (mean ± SD).

Fatty Acid	Batches				*p* Value
	CON	BHT	SSRE	MSRE	
C14:0	1107 ± 19.04	1113 ± 31.41	1110 ± 14.14	1095 ± 18.71	0.5042
C15:0	35.00 ± 10.49	32.67 ± 3.27	35.50 ± 5.01	30.00 ± 2.76	0.4166
C16:0	27,397 ± 138.70	27,349 ± 80.52	27,380 ± 33.47	27,265 ± 97.31	0.1120
C16:1	1380 ± 48.58	1384 ± 32.00	1370 ± 23.66	1365 ± 24.29	0.7457
C17:0	266.7 ± 15.06	260.0 ± 38.99	265.0 ± 22.58	265.0 ± 18.71	0.9718
C18:0	15,093 ± 158.80	14,982 ± 33.12	15,085 ± 69.50	15,092 ± 74.94	0.1583
C18:1cis-9	43,542 ± 237.90	43,627 ± 159.00	43,510 ± 137.00	43,598 ± 158.20	0.6631
C18:2n-6	10,105 ± 122.60 ^a^	10,192 ± 68.82 ^ab^	10,275 ± 86.89 ^b^	10,292 ± 59.47^b^	0.0053
C18:3n-3	368.3 ± 19.41	380.0 ± 14.14	375.0 ± 28.11	375.0 ± 30.82	0.8692
C20:0	213.3 ± 20.66	208.3 ± 14.38	205.0 ± 18.71	225.0 ± 27.39	0.3878
C20:2	366.7 ± 13.66	365.0 ± 17.61	345.0 ± 18.71	365.0 ± 24.29	0.1843
C20:3n-3	35.83 ± 4.36	39.17 ± 2.64	39.67 ± 2.87	40.00 ± 2.45	0.1197
SFA	44,112 ± 282.00	43,945 ± 83.34	44,081 ± 60.51	43,972 ± 129.60	0.2478
MUFA	44,972 ± 198.10	45,086 ± 130.90	44,880 ± 131.30	44,963 ± 166.80	0.2009
PUFA	10,876 ± 126.20 ^a^	10,976 ± 60.62 ^ab^	11,035 ± 100.50 ^b^	11,072 ± 73.23 ^b^	0.0096
n-6	10,105 ± 122.60 ^a^	10,192 ± 68.82 ^ab^	10,275 ± 86.89 ^b^	10,292 ± 59.47 ^b^	0.0053
n-3	404.2 ± 17.75	419.2 ± 15.84	414.7 ± 28.18	415.0 ± 32.19	0.7481
n-6/n-3	25.05 ± 1.27	24.35 ± 1.00	24.87 ± 1.54	24.93 ± 1.97	0.8525
PUFA/SFA	0.2450 ± 0.005 ^a^	0.2498 ± 0.001 ^ab^	0.2503 ± 0.002 ^b^	0.2518 ± 0.001 ^b^	0.0064
AI	0.5737 ± 0.005	0.5710 ± 0.003	0.5726 ± 0.001	0.5684 ± 0.004	0.1291
TI	1.516 ± 0.016	1.503 ± 0.005	1.512 ± 0.005	1.505 ± 0.006	0.0841
HH	1.897 ± 0.019	1.907 ± 0.008	1.901 ± 0.004	1.913 ± 0.011	0.1115

CON—control pâtés; BHT—pâtés with butylated hydroxytoluene added; SSRE—pâtés with sunflower stalk ethanol extract added; MSRE—pâtés with maize stalk ethanol extract added; AI—Atherogenic index; TI—Thrombogenic index; and HH—Hypocholesterolemic/hypercholesterolemic; ^a,b^ Different superscripts within the same row indicate a significant difference (*p* < 0.05); n = 3 for each group of pork liver pâtés in triplicate.

**Table 6:** The water activity (a_w_) of pork liver pâtés with added synthetic antioxidant and crop residue extracts (mean ± SD).

a_w_	Batches				Group *p* Value
	CON	BHT	SSRE	MSRE	
0 day	0.946 ± 0.001 ^aA^	0.946 ± 0.001 ^aA^	0.941 ± 0.001 ^bA^	0.947 ± 0.0001 ^aA^	<0.0001
20 day	0.956 ± 0.003 ^B^	0.959 ± 0.001 ^B^	0.957 ± 0.005 ^B^	0.958 ± 0.005 ^B^	0.4577
40 day	0.935 ± 0.001 ^aC^	0.950 ± 0.010 ^bA^	0.951 ± 0.007 ^bcB^	0.962 ± 0.005 ^cB^	<0.0001
60 day	0.921 ± 0.001 ^aD^	0.926 ± 0.001 ^bC^	0.923 ± 0.001 ^aC^	0.926 ± 0.001 ^bC^	<0.0001
90 day	0.902 ± 0.003 ^aE^	0.906 ± 0.001 ^bD^	0.910 ± 0.001 ^cD^	0.910 ± 0.001 ^cD^	<0.0001
Time *p* Value	<0.0001	<0.0001	<0.0001	<0.0001	
Group × Time *p* Value	<0.0001	<0.0001	<0.0001	<0.0001	

CON—control pâtés; BHT—pâtés with butylated hydroxytoluene added; SSRE—pâtés with sunflower stalk ethanol extract added; and MSRE—pâtés with maize stalk ethanol extract added; ^a,b,c^, Different lower-case superscript letters within the row indicate a significant difference between groups on the same day of storage (*p <* 0.05); ^A,B,C,D,E^ Different upper-case superscript letters within the column indicate differences within the same group on different days of storage (*p <* 0.05); n = 3 for each group of pork liver pâtés in triplicate.

**Table 7:** Physicochemical properties of pork liver pâtés during refrigerated storage (mean ± SD).

Days of Storage	Batches	Parameters					
		L*	a*	b*	C*	h°	pH
0							
	CON	56.05 ± 1.71 ^aA^	12.43 ± 0.66 ^a^	12.15 ± 0.75 ^a^	17.39 ± 0.77 ^aA^	44.38 ± 2.27 ^aAB^	6.48 ± 0.02 ^aAB^
	BHT	53.68 ± 4.45 ^aA^	13.24 ± 1.38 ^bABC^	12.56 ± 0.91 ^aA^	18.26 ± 1.45 ^bAC^	44.05 ± 1.16 ^aA^	6.46 ± 0.02 ^bA^
	SSRE	50.92 ± 2.86 ^bA^	12.89 ± 0.57 ^abA^	11.89 ± 0.39 ^aA^	17.46 ± 0.53 ^abA^	42.45 ± 1.46 ^aA^	6.42 ± 0.01 ^cA^
	MSRE	55.87 ± 2.70 ^aAB^	11.22 ± 0.61 ^cA^	14.08 ± 1.22 ^bA^	18.04 ± 0.83 ^abA^	51.36 ± 3.71 ^bA^	6.42 ± 0.01 ^cA^
20							
	CON	59.00 ± 1.71 ^aB^	12.90 ± 0.34 ^ac^	12.31 ± 0.44 ^a^	17.96 ± 0.69 ^aAB^	43.66 ± 0.76 ^aA^	6.50 ± 0.01 ^aB^
	BHT	58.09 ± 1.43 ^aB^	12.80 ± 0.56 ^aAB^	11.23 ± 0.33 ^bB^	17.04 ± 0.41 ^bBC^	41.30 ± 1.73 ^bB^	6.53 ± 0.01 ^bB^
	SSRE	52.17 ± 3.48 ^bA^	13.22 ± 0.36 ^cA^	11.95 ± 0.52 ^aAC^	17.73 ± 0.74 ^aA^	42.10 ± 0.93 ^abA^	6.45 ± 0.01 ^cBD^
	MSRE	57.18 ± 2.02 ^aAB^	10.86 ± 0.49 ^bA^	14.70 ± 1.05 ^cA^	18.30 ± 0.84 ^aA^	53.46 ± 3.01 ^cA^	6.49 ± 0.01 ^dB^
40							
	CON	54.03 ± 3.90 ^aA^	12.54 ± 0.70 ^a^	12.66 ± 0.67 ^a^	17.73 ± 0.74 ^aAB^	45.27 ± 1.77 ^aAB^	6.43 ± 0.01 ^aC^
	BHT	56.36 ± 3.52 ^abAB^	12.56 ± 0.34 ^aA^	11.26 ± 0.37 ^bB^	16.87 ± 0.41 ^bB^	41.88 ± 1.47 ^bB^	6.47 ± 0.01 ^bA^
	SSRE	57.57 ± 0.84 ^bB^	14.26 ± 0.49 ^bB^	12.84 ± 1.00 ^aB^	19.19 ± 1.09 ^cB^	41.93 ± 1.25 ^bA^	6.33 ± 0.06 ^cC^
	MSRE	57.91 ± 3.33 ^bA^	11.24 ± 0.47 ^cA^	14.13 ± 0.57 ^cA^	18.06 ± 0.57 ^aA^	51.48 ± 1.37 ^cA^	6.39 ± 0.01 ^dC^
60							
	CON	59.48 ± 3.46 ^aB^	12.70 ± 0.52 ^a^	12.34 ± 0.39 ^a^	17.71 ± 0.48 ^aAB^	44.16 ± 1.58 ^aAB^	6.49 ± 0.04 ^acB^
	BHT	57.57 ± 2.06 ^acB^	13.37 ± 0.51 ^bBC^	11.62 ± 0.46 ^bB^	17.69 ± 0.55 ^aC^	40.99 ± 1.46 ^bB^	6.51 ± 0.05 ^aC^
	SSRE	54.45 ± 1.82 ^bC^	14.01 ± 0.58 ^cB^	12.49 ± 0.44 ^aBC^	18.72 ± 0.75 ^bB^	41.49 ± 1.15 ^bA^	6.46 ± 0.01 ^bD^
	MSRE	55.54 ± 1.82 ^bcB^	12.58 ± 0.50 ^aB^	14.29 ± 0.52 ^cA^	19.03 ± 0.69 ^bB^	48.67 ± 0.79 ^cB^	6.47 ± 0.01 ^bcD^
90							
	CON	59.36 ± 3.57 ^aB^	12.53 ± 0.55 ^a^	12.71 ± 0.76 ^a^	18.08 ± 0.90 ^aB^	45.92 ± 3.19 ^aB^	6.49 ± 0.01 ^aB^
	BHT	55.06 ± 2.64 ^bA^	13.68 ± 0.59 ^bC^	13.77 ± 0.74 ^bC^	19.41 ± 0.82 ^bD^	45.18 ± 1.38 ^abA^	6.52 ± 0.01 ^bBC^
	SSRE	51.51 ± 1.46 ^cA^	14.79 ± 0.39 ^cC^	14.34 ± 0.37 ^bD^	20.60 ± 0.37 ^cC^	44.11 ± 1.08 ^bB^	6.43 ± 0.01 ^cAB^
	MSRE	55.56 ± 1.70 ^bB^	12.13 ± 0.41 ^aB^	15.87 ± 1.01 ^cB^	19.98 ± 0.94 ^bcC^	52.55 ± 1.55 ^cA^	6.44 ± 0.01 ^dE^
Group	*p* Value	<0.0001	<0.0001	<0.0001	<0.0001	<0.0001	<0.0001
Time	*p* Value	<0.0001	<0.0001	<0.0001	<0.0001	<0.0001	<0.0001
Group × Time	*p* Value	<0.0001	<0.0001	<0.0001	<0.0001	<0.0001	<0.0001
		ΔE 0		ΔE 90		ΔE*	
	CON	-		-		3.35	
	BHT	2.54		4.57		1.89	
	SSRE	5.16		8.33		3.15	
	MSRE	2.29		4.96		2.03	

CON—control pâtés; BHT—pâtés with butylated hydroxytoluene added; SSRE—pâtés with sunflower stalk ethanol extract added; MSRE—pâtés with maize stalk ethanol extract added; ΔE 0 and 90-color differences with respect to control pâtés on days 0 and 90; and ΔE*—color differences within the same group on days 0 and 90; ^a,b,c,d^ Different lower-case superscript letters within the column indicate a significant difference between groups on the same day of storage (*p <* 0.05); ^A,B,C,D,E^ Different upper-case superscript letters within the column indicate differences within the same group on different days of storage (*p <* 0.05); n = 3 for each group of pork liver pâtés in triplicate on every day of storage.

**Table 8:** Evolution of TBARS and peroxide values of pork liver pâté formulations during refrigerated storage time (mean ± SD).

Days of Storage	Batches	Parameters	
		POV (mmol/kg)	TBARs (MDA mg/kg)
0			
	CON	0.312 ± 0.053 ^aA^	0.088 ± 0.012 ^aA^
	BHT	0.018 ± 0.005 ^bA^	0.015 ± 0.004 ^bA^
	SSRE	1.442 ± 0.085 ^cA^	0.088 ± 0.010 ^aA^
	MSRE	1.665 ± 0.143 ^dA^	0.045 ± 0.010 ^cA^
20			
	CON	0.421 ± 0.058 ^aA^	0.090 ± 0.007 ^aA^
	BHT	0.183 ± 0.031 ^bB^	0.088 ± 0.007 ^aB^
	SSRE	2.355 ± 0.119 ^cB^	0.195 ± 0.007 ^bB^
	MSRE	2.345 ± 0.051 ^cB^	0.145 ± 0.009 ^cB^
40			
	CON	0.515 ± 0.093 ^aA^	0.135 ± 0.011 ^aB^
	BHT	0.474 ± 0.123 ^aC^	0.125 ± 0.004 ^bC^
	SSRE	2.660 ± 0.597 ^bB^	0.225 ± 0.011 ^cC^
	MSRE	2.500 ± 0.071 ^bC^	0.195 ± 0.012 ^dC^
60			
	CON	2.045 ± 0.469 ^aB^	0.145 ± 0.004 ^aB^
	BHT	1.175 ± 0.059 ^bD^	0.130 ± 0.008 ^aC^
	SSRE	4.335 ± 0.607 ^cC^	0.275 ± 0.011 ^bD^
	MSRE	2.645 ± 0.174 ^dD^	0.205 ± 0.038 ^cC^
90			
	CON	1.275 ± 0.152 ^aC^	0.340 ± 0.044 ^acC^
	BHT	1.142 ± 0.088 ^bD^	0.195 ± 0.027 ^bD^
	SSRE	2.515 ± 0.120 ^cB^	0.450 ± 0.021 ^cE^
	MSRE	1.195 ± 0.048 ^aE^	0.345 ± 0.033 ^aD^
Group	*p* Value	<0.0001	<0.0001
Time	*p* Value	<0.0001	<0.0001
Group × Time	*p* Value	<0.0001	<0.0001

CON—control pâtés; BHT—pâtés with butylated hydroxytoluene added; SSRE—pâtés with sunflower stalk ethanol extract added; and MSRE—pâtés with maize stalk ethanol extract added; ^a,b,c,d^ Different lower-case superscript letters within the column indicate a significant difference between groups on the same day of storage (*p <* 0.05); ^A,B,C,D,E^ Different upper-case superscript letters within the column indicate differences within the same group on different days of storage (*p <* 0.05); n = 3 for each group of pork liver pâtés in triplicate on every day of storage.

**Table 9:** Microbial counts (total viable count (TVC), lactic acid bacteria (LAB), and psychrotrophic bacteria) in pork liver pâtés during chilling storage (log CFU/g) (mean ± SD).

Days of Storage	Batches	Microbial Counts		
		TVC	LAB	Psychrotrophic
0				
	CON	1.80 ± 0.19 ^aA^	1.53 ± 0.13 ^aA^	1.51 ± 0.14 ^aA^
	BHT	1.75 ± 0.21 ^aA^	1.60 ± 0.11 ^aA^	1.40 ± 0.16 ^abA^
	SSRE	1.47 ± 0.17 ^bA^	1.41 ± 0.13 ^bA^	1.30 ± 0.11 ^bA^
	MSRE	1.55 ± 0.15 ^bA^	1.35 ± 0.11 ^bA^	1.36 ± 0.13 ^bA^
20				
	CON	2.37 ± 0.15 ^aB^	2.34 ± 0.13 ^aB^	2.20 ± 0.14 ^B^
	BHT	2.44 ± 0.14 ^aB^	2.22 ± 0.13 ^acB^	2.24 ± 0.12 ^B^
	SSRE	2.19 ± 0.09 ^bB^	2.01 ± 0.27 ^bB^	2.15 ± 0.10 ^B^
	MSRE	2.21 ± 0.08 ^bB^	2.10 ± 0.28 ^bcB^	2.14 ± 0.14 ^B^
40				
	CON	3.37 ± 0.10 ^aC^	2.96 ± 0.44 ^aC^	3.18 ± 0.08 ^aC^
	BHT	3.44 ± 0.14 ^aC^	2.74 ± 0.32 ^abC^	3.10 ± 0.11 ^abC^
	SSRE	3.12 ± 0.13 ^bC^	2.56 ± 0.33 ^bC^	3.03 ± 0.09 ^bC^
	MSRE	3.20 ± 0.12 ^bC^	2.70 ± 0.37 ^abC^	3.03 ± 0.10 ^bC^
60				
	CON	4.52 ± 0.14 ^aD^	4.22 ± 0.26 ^aD^	4.10 ± 0.19 ^aD^
	BHT	4.41 ± 0.09 ^bD^	3.95 ± 0.24 ^bD^	4.00 ± 0.11 ^aD^
	SSRE	3.98 ± 0.14 ^cD^	3.75 ± 0.33 ^bD^	3.80 ± 0.11 ^bD^
	MSRE	3.81 ± 0.06 ^dD^	3.70 ± 0.32 ^bD^	3.83 ± 0.13 ^bD^
90				
	CON	5.05 ± 0.16 ^aE^	4.71 ± 0.19 ^aE^	4.60 ± 0.17 ^aE^
	BHT	5.11 ± 0.15 ^aE^	4.63 ± 0.22 ^aE^	4.49 ± 0.14 ^aE^
	SSRE	4.69 ± 0.16 ^bE^	4.14 ± 0.17 ^bE^	4.00 ± 0.14 ^bE^
	MSRE	4.73 ± 0.11 ^bE^	4.16 ± 0.24 ^bE^	4.07 ± 0.14 ^bE^
Group	*p* Value	<0.0001	<0.0001	<0.0001
Time	*p* Value	<0.0001	<0.0001	<0.0001
Group × Time	*p* Value	<0.0001	<0.0001	<0.0001

CON—control pâtés; BHT—pâtés with butylated hydroxytoluene added; SSRE—pâtés with sunflower stalk ethanol extract added; and MSRE—pâtés with maize stalk ethanol extract added; ^a,b,c,d^ Different lower-case superscript letters within the column indicate a significant difference between groups on the same day of storage (*p <* 0.05); ^A,B,C,D,E^ Different upper-case superscript letters within the column indicate differences within the same group on different days of storage (*p <* 0.05); n = 3 for each group of pork liver pâtés in triplicate on every day of storage.

## Data Availability

The original contributions presented in the study are included in the article, further inquiries can be directed to the corresponding author.
